# Exploring brusatol as a new anti-pancreatic cancer adjuvant: biological evaluation and mechanistic studies

**DOI:** 10.18632/oncotarget.17761

**Published:** 2017-05-10

**Authors:** Zheng Lu, Zheng-Quan Lai, Albert W.N. Leung, Po Sing Leung, Zhao-Shen Li, Zhi-Xiu Lin

**Affiliations:** ^1^ School of Chinese Medicine, Faculty of Medicine, The Chinese University of Hong Kong, Shatin, China; ^2^ Department of Gastroenterology, Changhai Hospital, The Second Military Medical University, Shanghai, China; ^3^ School of Biomedical Sciences, Faculty of Medicine, The Chinese University of Hong Kong, Shatin, China; ^4^ Liver Cirrhosis Diagnosis and Treatment Center, Beijing 302 Hospital, Beijing, China

**Keywords:** pancreatic cancer, brusatol, gemcitabine, 5-fluorouracil, combination therapy

## Abstract

Pancreatic cancer is highly resistant to chemotherapeutic agents and is known to have a poor prognosis. The development of new therapeutic entities is badly needed for this deadly malignancy. In this study, we demonstrated for the first time that brusatol, a natural quassinoid isolated from a Chinese herbal medicine named Bruceae Fructus, possessed potent cytotoxic effect against different pancreatic adenocarcinoma cell lines. Its anti-pancreatic cancer effect was comparable to that of the first-line chemotherapeutic agents such as gemcitabine and 5-fluorouracil, with a more favorable safety profile. In addition, brusatol showed a synergistic anti-proliferative effect toward PANC-1 and Capan-2 cell lines when combined with gemcitabine or 5-fluorouracil. The results of flow cytometry suggested that brusatol combination treatment with gemcitabine or 5-fluorouracil was able to cause cell cycle arrest at G2/M phase, and accentuate apoptosis in PANC-1 cells. Moreover, brusatol deactivated gemcitabine/5-fluorouracil-induced NF-κB activation. Western blot analysis and qRT-PCR results showed that brusatol significantly down-regulated the expression of vimentin and Twist, and markedly stimulated the expression of E-cadherin, the key regulatory factors of the epithelial-mesenchymal transition process. Furthermore, treatment with combination of brusatol and gemcitabine or 5-fluorouracil significantly reduced *in vivo* tumor growth when compared with treatment of either brusatol or gemcitabine/5-fluorouracil alone. Taken together, these results have amply demonstrated that brusatol is a potent anti-pancreatic cancer natural compound, and the synergistic anti-pancreatic cancer effects of brusatol and gemcitabine/5-fluorouracil observed both *in vitro* and *in vivo* are associated with the suppression of epithelial-mesenchymal transition process, indicating that brusatol is a promising adjunct to the current chemotherapeutic regimen.

## INTRODUCTION

Pancreatic cancer (PanCa), one of the most deadly human malignancies, has a 5-year survival rate of less than 1%. PanCa is the 4^th^ most common cause of the cancer-related death in the USA [1], where in 2014, about 46,420 people were diagnosed with PanCa and approximately 39,590 people died of this disease [2]. In China, the past two decades have witnessed a 6-time increase in the incidence rate of PanCa, possibly owing to the increasing prevalence of Western diets-induced obesity. Today, PanCa has become the 6^th^ leading cause of cancer-related mortality in China [3].

Currently, pharmacological treatment using gemcitabine (GEM) and 5-fluorouracil (5-FU) is among the most common strategies for the treatment of advanced and unresectable PanCa. However, the median survival time is still less than 6 months for patients on these treatments [4], due mainly to drug resistance [5]. To overcome chemo-resistance, combination chemotherapy strategies are usually adopted to achieve better cancer cell killing with fewer systemic toxicity [6]. To enhance the efficacy of current chemotherapeutic agents such as GEM and 5-FU for PanCa, new adjuvants which can circumvent GEM/5-FU-associated chemo-resistance are badly needed.

In recent years, herbal medicines or natural compounds, either used alone or combined with conventional chemotherapeutic agents, have been shown to have beneficial effects on diverse cancers [7]. In our previous work, we found that the alcoholic extract of Bruceae Fructus (*Ya-Dan-Zi* in Chinese), a Chinese medicinal herb commonly used for the treatment of cancer, possessed significant cytotoxicity against several PanCa cell lines [8]. Brucein D, a quassinoid found in abundance in this herb, has been shown to inhibit the activity of NF-κB and suppress the growth of several PanCa cell lines via inducing cellular apoptosis without causing overt organ toxicity [9,10]. Brusatol (BR), a natural quassinoid diterpenoid isolated from Bruceae Fructus, exhibited the most potent *in vitro* anti-pancreatic tumor action among all the isolated quassinoids [11]. Furthermore, it was reported that brusatol acted as a unique inhibitor of the Nrf2 pathway that sensitized various cancer cells and A549 xenografts to chemotherapeutic drugs, suggesting brusatol might be a promising candidate for combating chemo-resistance and has the potential to be developed into an adjuvant chemotherapeutic agent [12]. However, the chemosensitizing effect of brusatol on PanCa has not been explored.

It has been known that epithelial-mesenchymal transition (EMT) involving key regulators such as Twist and E-cadherin, is an important mechanism underlying chemotherapy resistance [13,14]. Targeted inhibition of the EMT process without eliciting systemic toxicity using combination chemotherapeutic agents may lead to better tumor cell killing in PanCa. Based on the anti-PanCa and chemosensitizing effect of brusatol, we hypothesized that brusatol could sensitize the current first-line chemotherapeutic agents GEM and 5-FU to PanCa via inhibition on the EMT process. This work was therefore initiated to explore the potential of brusatol as a novel anti-PanCa adjuvant for GEM and 5-FU using different PanCa cell lines *in vitro*, and *in vivo* via orthotopic xenotransplantation PanCa mice model. Moreover, the underlying molecular mechanisms were delineated. Our results indicated that brusatol deactivated NF-κB activation and arrested PanCa cell growth at least in part via inhibition of Twist and stimulation of E-cadherin expression. The synergistic inhibition by brusatol and GEM or 5-FU observed both *in vitro* and *in vivo* suggested that brusatol may be a promising adjunct to current chemotherapeutic regimens.

## RESULTS

### Brusatol inhibits proliferation and potentiates the inhibitory effects of chemotherapeutic agents in PanCa cells

**Figure 1:**
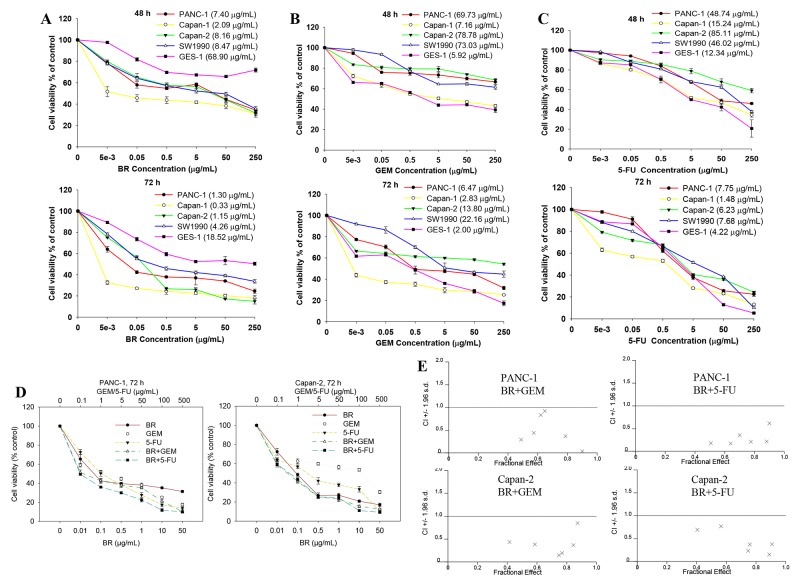
Brusatol (BR) inhibits proliferation and potentiates the inhibitory effects of chemotherapeutic agents in PanCa cells **(A-C)** Cytotoxic effects of brusatol (A), GEM (B) and 5-FU (C) on the growth of human PanCa cell lines, and non-tumorigenic human gastric cell line (GES-1). IC_50_ values were shown in parentheses immediately after the names of the cell lines in the graphic legends. **(D)** Cell growth inhibition elicited by GEM, 5-FU and the combination with brusatol in PANC-1 and Capan-2 cells as measured by MTT assay. **(E)** Fa-CI plots for combination treatment with brusatol and GEM or 5-FU in PANC-1 and Capan-2 cells. Combination index (CI) is a quantitative measurement of the degree of drug interaction. CI < 1 indicates synergism, CI = 1 indicates additive effect, while CI > 1 signifies antagonism. × represents that CI values were generated over a range of 40%-95% growth inhibitory effects.

The cytotoxic effects of brusatol, GEM and 5-FU on the cell viability of human PanCa cell lines PANC-1, Capan-1, Capan-2, SW1990, and the non-tumorigenic human gastric cell line (GES-1) are shown in Figure 1A-1C. The results showed that brusatol markedly suppressed the cell proliferation of all the four tested PanCa cell lines in a dose- and time-dependent manner, with IC_50_ values in the range of 0.33-8.47 μg/mL. The potency of brusatol was significantly higher than those of GEM (IC_50_: 2.83-78.78 μg/mL) and 5-FU (IC_50_: 1.48-85.11 μg/mL). However, brusatol only exerted mild cytotoxicity on GES-1 cells, with IC_50_ value > 68.90 μg/mL. In contrast, GEM and 5-FU were more toxic to GES-1 cells, with IC_50_ values being 5.92 μg/mL and 12.34 μg/mL, respectively. These results unambiguously indicated that brusatol possessed potent *in vitro* anti-PanCa effect.

For single-agent treatment, GEM or 5-FU (0.1, 1, 5, 10, 100, 500 μg/mL) alone produced a dose-dependent inhibition on the growth of PANC-1 and Capan-2 cells when cultured for 72 h. When GEM or 5-FU was combined with brusatol at a constant concentration ratio of 10:1, by adding 0.01, 0.1, 0.5, 1, 10 and 50 μg/mL brusatol, respectively, the cell growth inhibition was greatly enhanced (Figure 1D). The combination-index (CI) is a mathematical method commonly used to measure the pharmacological interaction of two drugs [15-18]. Isobologram analysis showed that the CI for every combination treatment was < 1 (Figure 1E and Supplementary Table 1), indicating significant synergistic effects of these combination treatments. It is clear from the results that combination of GEM or 5-FU with lower doses of brusatol elicited significantly greater inhibition on the cancer cell growth than either agent alone.

Since brusatol showed a synergistic anti-proliferative effect when combined with GEM or 5-FU in PANC-1 and Capan-2 cell lines, and PANC-1 and Capan-2 cells were more sensitive to the combination treatment and relatively easier to culture, they were therefore used for the subsequent mechanistic studies.

### Brusatol induces apoptosis and causes G2/M cell cycle arrest in PanCa cells

**Figure 2:**
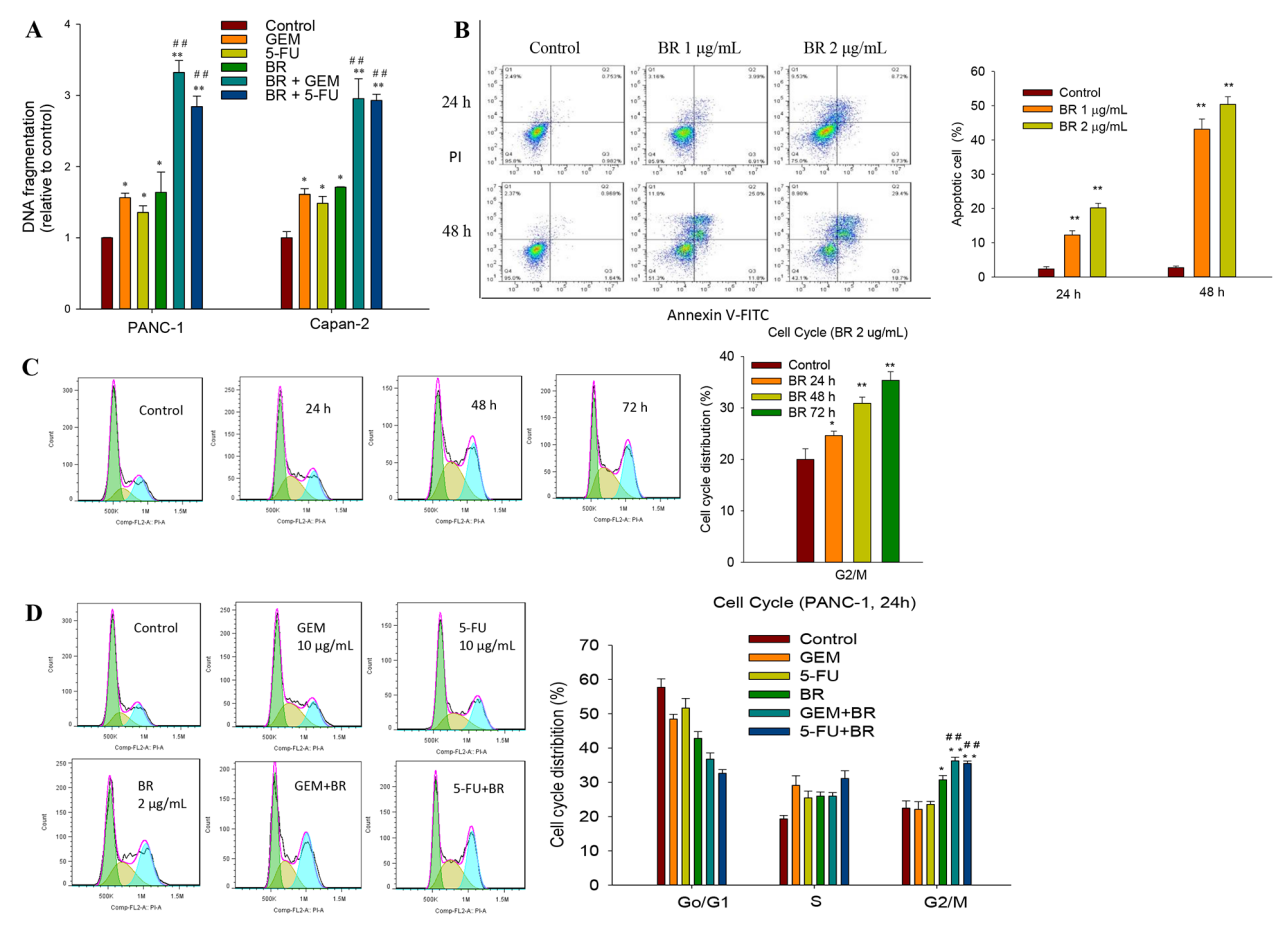
Brusatol induces apoptosis and causes G2/M cell cycle arrest in PanCa cells **(A)** Quantitative measurement of DNA fragmentation in PANC-1 and Capan-2 cells using Cell Death Detection ELISA^PLUS^ Kit. **(B)** Brusatol-induced apoptosis in PANC-1 cells detected by Annexin V-PI stain assay. Criteria were set to distinguish between viable (bottom left), early apoptotic (bottom right), late apoptotic (top right) and necrotic (top left) cells. **(C)** Brusatol time-dependently arrested cell cycle at the G2/M phase as assessed by PI staining. **(D)** Combination treatment with brusatol and GEM or 5-FU significantly arrested cell cycle at the G2/M phase as assessed by PI staining at 24 h. The data in A-D are represented as the mean ± SEM; *P < 0.05, **P < 0.01 vs control and #*P* < 0.05, ##*P* < 0.01 vs chemotherapeutic agent treatment alone. All data are representative of three independent experiments.

Quantification of DNA fragmentation in PANC-1 and Capan-2 cells was performed using Cell Death Detection ELISA^PLUS^ Kit. Cells were treated with DMEM medium (control), GEM (10 μg/mL), 5-FU (10 μg/mL), brusatol (2 μg/mL) or combination treatment for 48 h. The results revealed that brusatol caused significant DNA fragmentation after exposure for 48 h. Noticeably, brusatol combined with GEM or 5-FU induced more cellular apoptosis than the GEM or 5-FU alone group (Figure 2A). Quantitative flow cytometric analysis showed that brusatol exerted a dose- and time-dependent apoptogenic effect on PANC-1 cells (Figure 2B). At 1 and 2 μg/mL, brusatol caused 10.9% and 15.5% of the cancer cells to undergo apoptosis within 24 h, respectively. Extending the treatment time to 48 h resulted in a marked augmentation of cellular apoptosis, with 36.8% and 48.1% of cellular apoptosis after 1 and 2 μg/mL brusatol treatments, respectively. In stark contrast, non-treated cells showed normal cell viability without significant cell death.

To further elucidate the mechanism of growth inhibition and whether the cell cycle change upon brusatol monotherapy and combination treatment, PANC-1 cells were treated with 2 μg/mL brusatol for 24, 48 and 72 h, and exposed to brusatol alone and in combination with GEM or 5-FU for 48 h. The results indicated that brusatol alone induced a time-dependent G2/M arrest (Figure 2C), and brusatol combined with GEM or 5-FU also resulted in a pronounced accumulation of cells in G2/M (Figure 2D).

### Brusatol treatment decreases the expression of anti-apoptotic protein and deactivates chemotherapeutic agents-induced NF-κB activation

**Figure 3:**
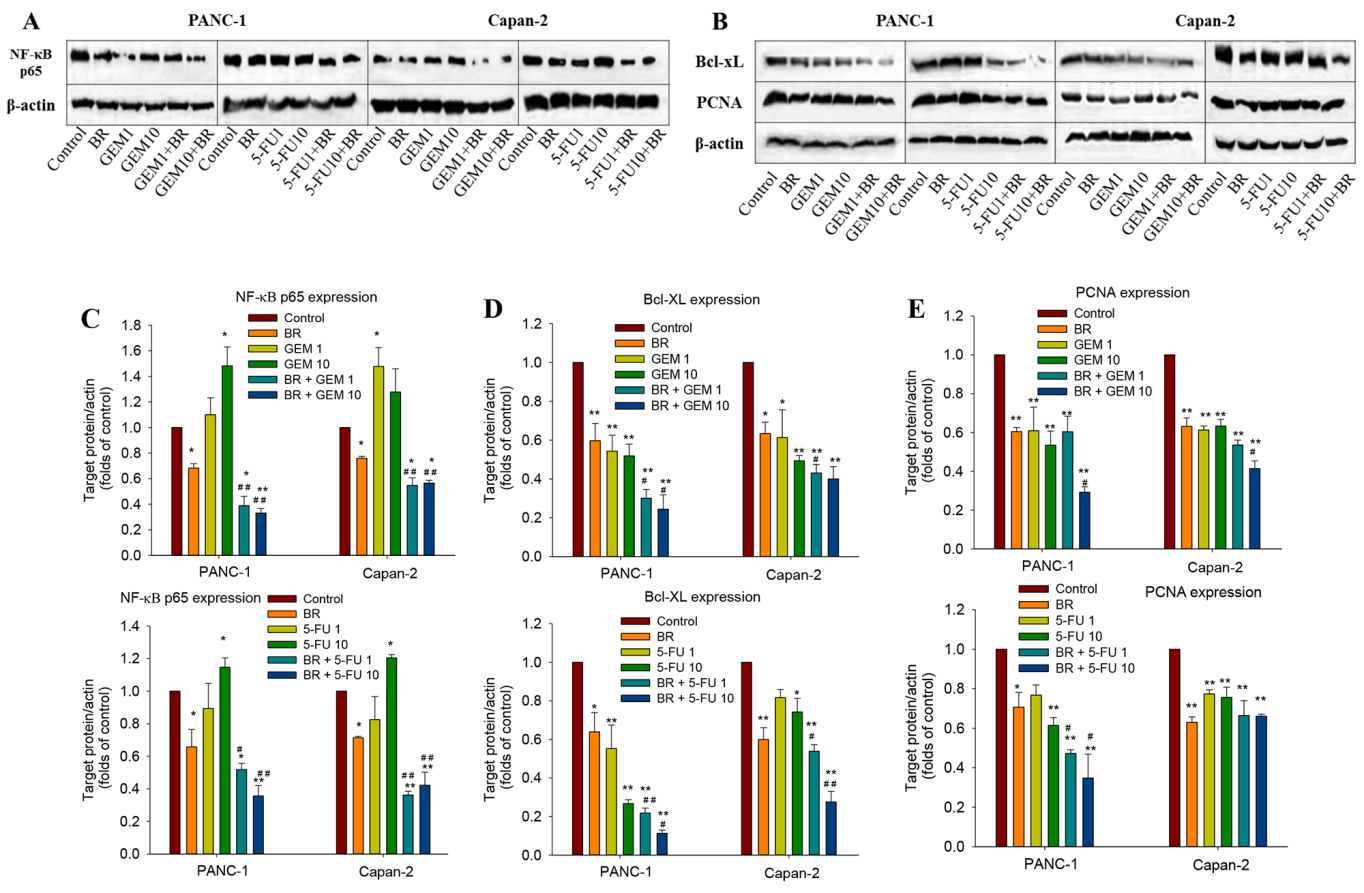
Brusatol deactivates chemotherapeutic agents-induced NF-κB activation and inhibits the anti-apoptotic protein in PanCa cells **(A-B)** Western blot analysis of the expression of NF-κB p65, Bcl-xL and PCNA in PANC-1 and Capan-2 cells. **(C-E)** Quantitative analysis of Western blot band intensities. The data in C-E are represented as the mean ± SEM; *P < 0.05, **P < 0.01 vs control and #*P* < 0.05, ##*P* < 0.01 vs chemotherapeutic agent treatment alone. All data are representative of three independent experiments.

As shown in Figure 3A and 3C, immunoblotting results clearly showed the down-regulation of NF-κB p65 in PanCa cells after exposure to brusatol, and the combined treatment with chemotherapeutic agents significantly augmented the brusatol-mediated inhibition of NF-κB p65. In addition, brusatol down-regulated the expression of two anti-apoptotic proteins Bcl-xL and PCNA in both PANC-1 and Capan-2 cells. Combination treatment of brusatol with GEM or 5-FU also markedly attenuated the expression of Bcl-xL and PCNA (Figure 3B, 3D and 3E). The immunoblotting results are strongly indicative that brusatol could suppress the expression of anti-apoptotic proteins in favor of promoting cellular apoptosis. These findings were congruent with cell growth inhibition as observed in MTT assay, amply vindicating that the greater cell growth inhibition observed with the combination treatment was closely associated with the induction of cellular apoptosis.

### Effects of brusatol on the EMT process in PanCa cells

**Figure 4:**
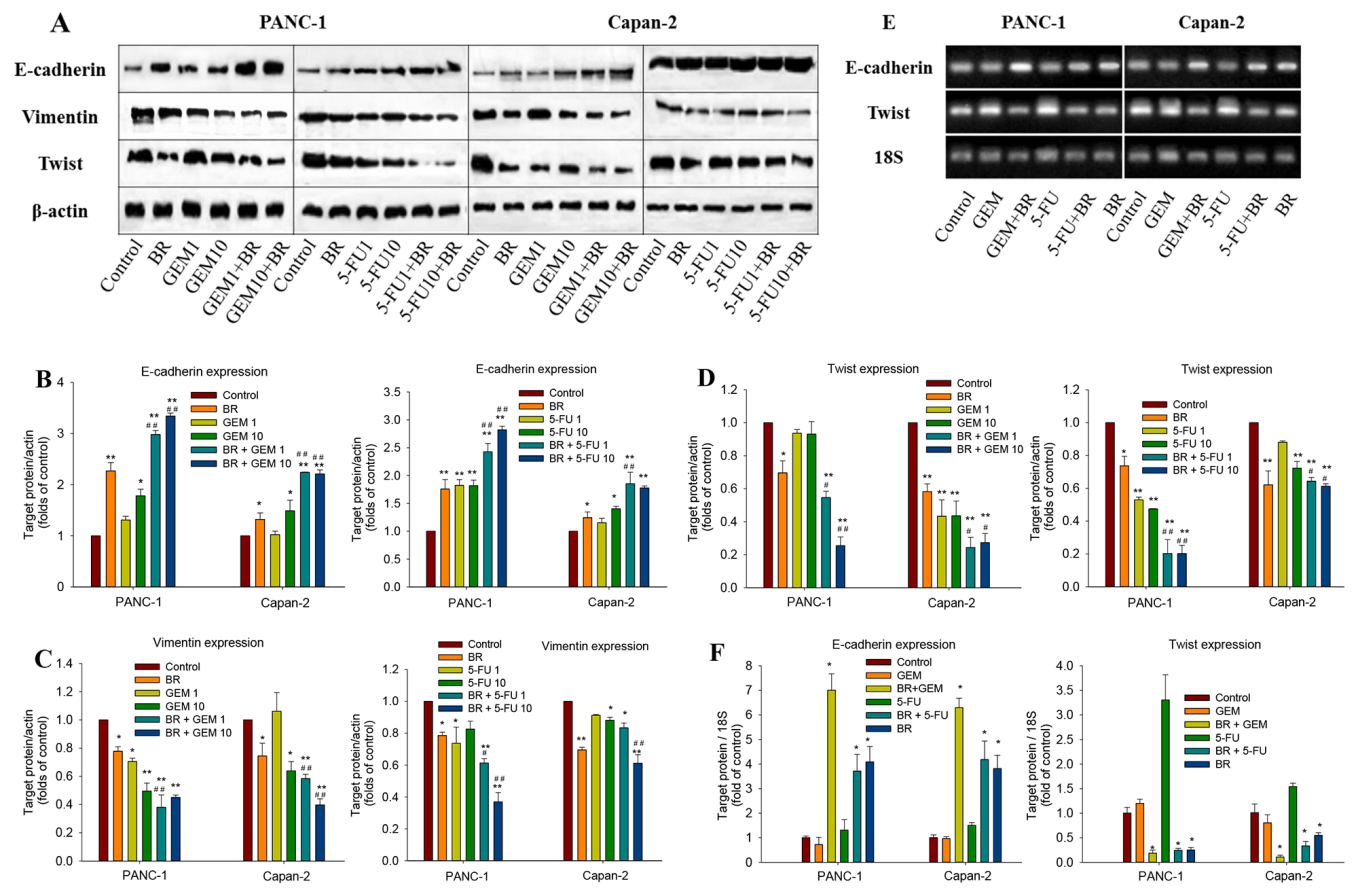
Effects of brusatol on the EMT process in PanCa cells **(A)** Western blot analysis of the expression of key regulatory factors (E-cadherin, vimentin, and Twist) of the EMT process in PANC-1 and Capan-2 cells. **(B-D)** Quantitative analyses of the Western blot band intensities. **(E)** qRT-PCR analysis of the mRNA expression of E-cadherin and Twist. The electrophoresis of RT-PCR product was amplified by 18S, E-cadherin and Twist primers. **(F)** Brusatol significantly increased E-cadherin mRNA expression and decreased Twist mRNA expression alone and in combination with GEM or 5-FU in both PANC-1 and Capan-2 cells. Expression of E-cadherin and Twist was normalized against the housekeeping gene 18S. The data in B-D and F are represented as the mean ± SEM; *P < 0.05, **P < 0.01 *vs* control, and #*P* < 0.05, ##*P <* 0.01 *vs* chemotherapeutic agent treatment alone. All data are representative of three independent experiments.

EMT is an important mechanism associated with chemoresistance. In the present work, the expression of E-cadherin, vimentin and Twist, three characteristic factors of the EMT process, was detected by Western blotting after brusatol alone or combination treatment for 48 h. The results showed that brusatol markedly increased the E-cadherin expression, while significantly decreased vimentin expression. As shown in Figure 4A and 4B, brusatol combined with chemotherapeutic agents induced stronger E-cadherin protein expression in PANC-1 cells, with 3.3-fold over that of GEM and 2.7-fold 5-FU, respectively. In contrast, the expression of vimentin (Figure 4A and 4C) and Twist (Figure 4A and 4D) decreased significantly after combination treatment when compared with the control.

Effects of brusatol on E-cadherin and Twist expression in PANC-1 and Capan-2 cells were further analyzed by real-time PCR. The results revealed the significantly increased E-cadherin mRNA expression and decreased Twist expression in both the brusatol monotherapy and combination treatments, as compared with the untreated control (Figure 4E and 4F). The results implied that brusatol alone or in combination with chemotherapeutic agents could increase the expression of E-cadherin, while suppress the expression of Twist and vimentin, thus inhibiting the EMT process, and ultimately leading to the chemosensitizing effect of brusatol.

### Brusatol and its combination with chemotherapeutic agents significantly inhibits the tumor growth in human pancreatic orthotopic xenograft tumor mouse model

Based on the promising *in vitro* results concerning the anti-PanCa effect of brusatol and its combination with GEM or 5-FU, we undertook the *in vivo* studies to investigate whether brusatol alone, or in combination with chemotherapeutic agents, could inhibit the growth of pancreatic tumor in an orthotopic animal model.

**Figure 5:**
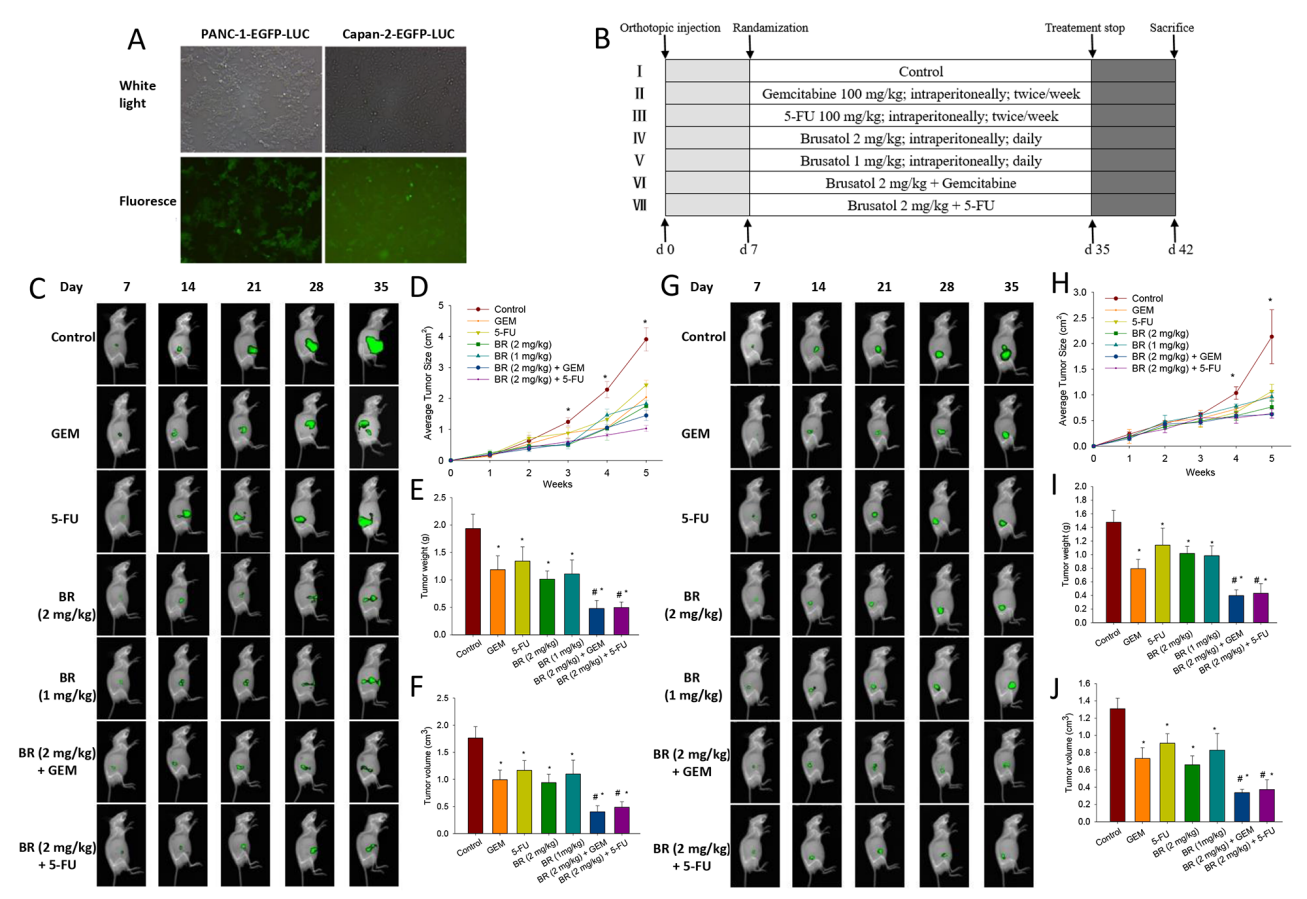
Brusatol and its combination with chemotherapeutic agents significantly inhibit the tumor growth in human pancreatic orthotopic xenograft mouse model **(A)** Expression of EGFP was observed by fluorescent microscopy. Lentivirus vector transfection produced up to 80% EGFP-positive cells after 14 days as screened by puromycin. **(B)** Schematic presentation of the experimental protocol described in the Materials and Methods. **(C-F)** Brusatol alone or in combination with chemotherapeutic agents markedly inhibited the tumor growth in PANC-1 orthotopic xenograft mouse model. Whole-body imaging, real-time analysis of pancreatic tumor growth was performed in live, anesthetized mice every week. Panels depict a representative mouse from each group (C). Quantification of orthotopic xenograft tumor luciferase fluorescence enabled real-time determination and comparison of tumor load during the course of treatment, thereby permitting real-time comparison of treatment efficacy between groups at various time points. Points are the mean areas of luciferase fluorescence for live intact animals in each group; error bars represent SEM (D). Tumor volumes measured on the last day of the experiment at autopsy using Vernier calipers and calculated using the formula V = (*a×b×c*)/2 (*n* = 5) (E). Tumor weight of each group on the last day of the experiment (*n* = 5) (F). **(G-J)**, Brusatol alone or in combination with chemotherapeutic agents markedly inhibited the tumor growth in Capan-2 orthotopic xenograft mouse model. Procedures for sequential whole-body imaging and quantification of tumor volume and weight were similar to those described in the C-F. *P < 0.05 vs control and #*P* < 0.05 vs chemotherapeutic agent treatment alone.

The lentivirus expressing EGFP and luciferase were used to infect PANC-1 and Capan-2 cells. We observed under a fluorescent microscope for the expression of EGFP at different time-points after infecting the cells with lentivirus and then calculated the infection efficiency. When the multiplicity of infection (MOI) was 100, the infection efficiency of lentivirus was above 80% after incubation for 72 h in PANC-1 and Capan-2 cells. Following 2-week screening by puromycin, the infection efficiency was maintained stably in the range of 80-90% (Figure 5A). The results of luciferase activity assay showed that lentivirus could steadily infect PANC-1 and Capan-2 cells and the infection efficiency was satisfactory. In addition, there was no significant difference in morphology, growth rate and tumor formation rate between parent and CMV-EGFP-linker-Luc-transfected cells (data not shown). Hence, the cell lines expressing both EGFP and luciferase could serve as a promising tool for real-time monitoring of tumor growth *in vivo*.

For the construction of orthotopic PanCa mouse model, PANC-1 and Capan-2 cells (stably transfected with EGFP and luciferase) were injected into the pancreas as described in the Methods. The experimental protocol is depicted in Figure 5B. As shown in Figure 5C and 5G, the luciferase fluorescence made possible the real-time and sequential whole-body imaging of tumors. Moreover, noninvasive quantitative measurements of the externally visible fluorescent area enabled the construction of the *in vivo* tumor growth curves (Figure 5D and 5H). Small primary tumor lesions on day 7 after transplantation were observed in all mice by the real-time whole-body imaging. The differences in imaging were not significant on day 14. However, imaging conducted on days 21, 28 and 35 confirmed the significant growth of primary tumor in the control group. While 1 or 2 mg/kg brusatol group, and its combination with GEM or 5-FU groups showed a marked reduction in the tumor growth in a dose-dependent manner when compared with the control. The size of xenograft tumors formed in mice of 2 mg/kg group was markedly smaller than that of the 1 mg/kg brusatol-treated mice. On days 28 and 35, in both PANC-1 and Capan-2 orthotopic xenograft tumor models, brusatol (1 or 2 mg/kg), and its combination with GEM or 5-FU were shown to significantly inhibit the tumor size as compared with the corresponding control (Figure 5C, 5D, 5G and 5H).

Figure 5E and 5I showed the effects of brusatol and chemotherapeutic agents on the tumor weight and volume as measured at the end of the experiment by autopsy. Results indicated that brusatol, GEM, 5-FU and their combinations all significantly reduced the tumor volume and weight as compared with the corresponding control in both orthotopic xenograft tumor models (Figure 5E, 5F, 5I and 5J). Brusatol at 1 mg/kg was also found to exhibit significant anti-tumor activity albeit the effect was less potent when compared with that of 2 mg/kg brusatol. The combined treatment of brusatol at 2 mg/kg with chemotherapeutic agents significantly reduced the tumor volume and weight, and the tumor reduction was more pronounced than either agent alone.

### *In vivo* toxicity test of brusatol

**Figure 6:**
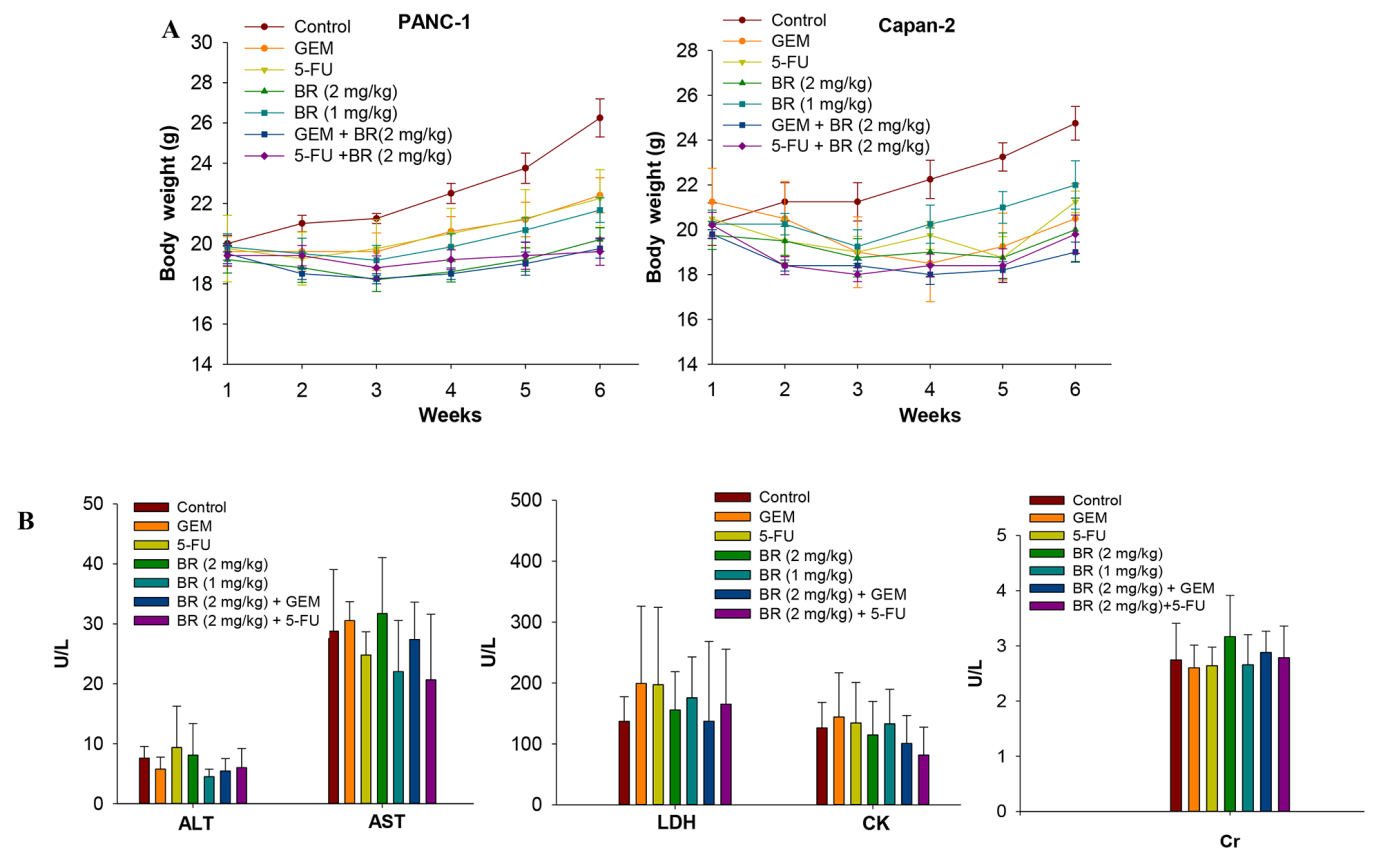
*In vivo* toxicity test of brusatol Effects of brusatol alone or in combination with chemotherapeutic agents on the body weight of PANC-1 and Capan-2 orthotopic xenograft nude mice within 6 weeks **(A)**, and plasma enzyme levels of alanine aminotransferase (ALT), aspartate aminotransferase (AST), lactate dehydrogenase (LDH), creatine kinase (CK) and creatinine (Cr) in Capan-2 orthotopic xenograft nude mice after treatment for 28 consecutive days **(B)**.

To evaluate the potential toxicity of brusatol, acute toxicity assessment was performed.Nude mice treated with brusatol intraperitoneally at the high dose (2 mg/kg) for 28 consecutive days did not show any treatment-related side effects and signs of toxicity. The body weight of the brusatol-treated and combination-treated nude mice showed no significant changes when compared to that of the GEM/5-FU alone treatment. A larger accrual in the body weights of the control mice observed might be caused by more rapid tumor growth in control group (Figure 6A). In addition, no significant differences were observed in the levels of plasma enzymes including ALT, AST, LDH, CK and Cr (Figure 6B) between the treatment groups and the control. Furthermore, brusatol induced no treatment-related abnormality concerning the gross anatomy and histological morphology. The results indicated that brusatol at the high dose of 2 mg/kg exerted no overt toxicity to the liver, heart and kidney tissues in the tumor-bearing mice.

### Expression of E-cadherin and Twist in the orthotopic xenograft tumor tissues

**Figure 7:**
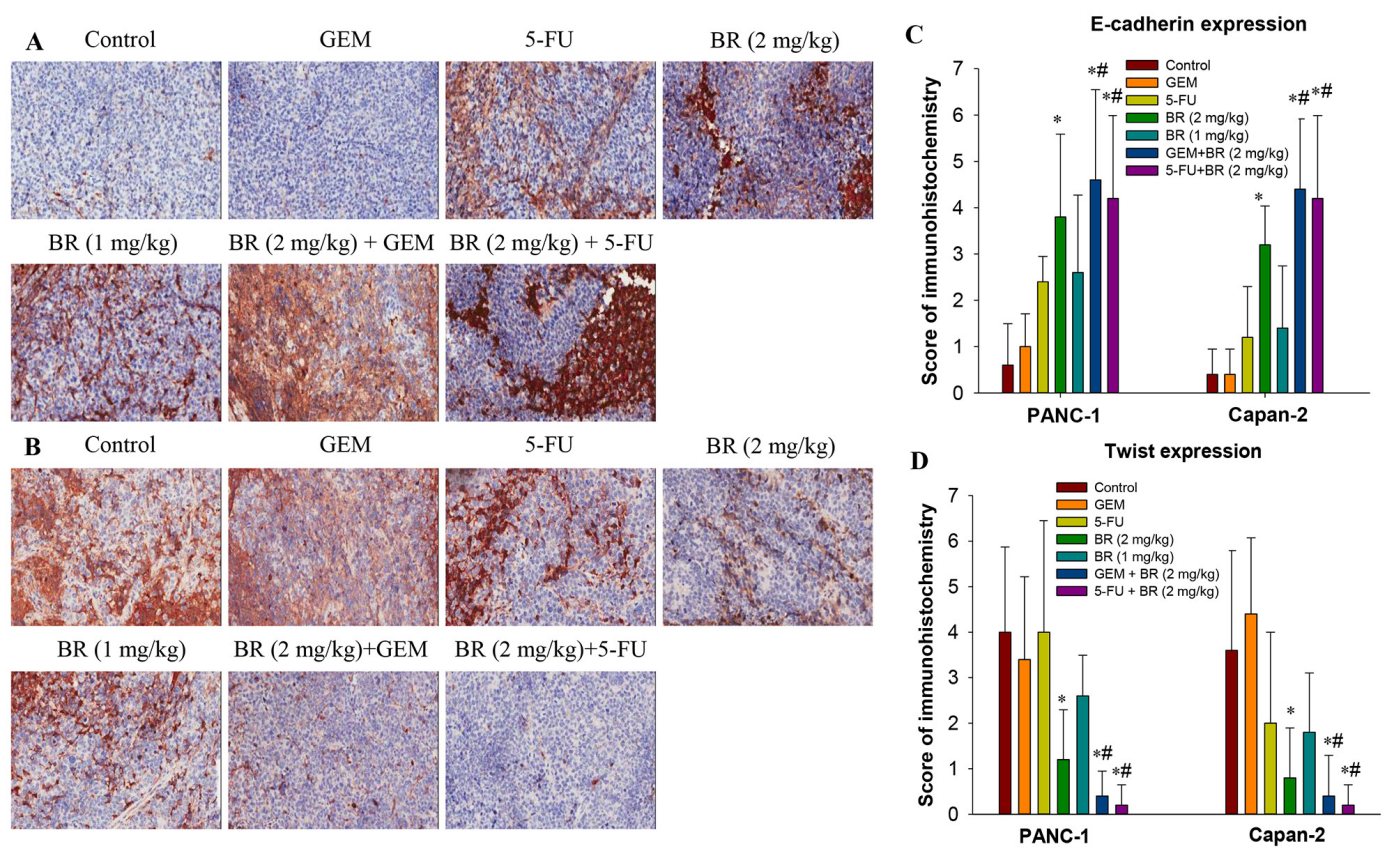
Expression of E-cadherin and Twist in the orthotopic xenograft tumor tissues by immunohistochemistry **(A-B)** Effects of brusatol on E-cadherin (A) and Twist (B) expression levels in PANC-1 xenograft tissues. **(C-D)** Scoring of E-cadherin and Twist expression. Each slide was scored semi-quantitatively on the basis of percentage and intensity of the stained normal or neoplastic epithelial cells. The total score was the product of the scores for the intensity and positive rate of the staining. Samples analyzed were from 5 animals in each group. All images were taken at 200× magnification. The data in C and D are represented as the mean ± SEM; *P < 0.05 vs control and #*P* < 0.05 vs chemotherapeutic agent treatment alone.

The Twist and E-cadherin expression levels in pancreatic tumor tissues were measured. We found that both brusatol and 5-FU alone groups upregulated the expression of E-cadherin in PANC-1 and Capan-2 orthotopic xenograft tumor tissues (Figure 7A-7D). The result confirmed our *in vitro* data that brusatol treatment upregulated the E-cadherin levels in PanCa cells. Similarly, we found that brusatol alone decreased the Twist expression, as compared with the control group. The differences in the expression of E-cadherin and Twist between different brusatol groups and the control group were statistically significant. In addition, in both PANC-1 and Capan-2 orthotopic xenograft tumor tissues, brusatol combined with GEM or 5-FU group showed stronger staining for E-cadherin, and weaker Twist staining when compared with the chemotherapeutic agent alone and the control group, and the combination treatment was observed to produce the most obvious effect (*P* < 0.01).

## DISCUSSION

Brusatol, a quassinoid found in abundance in Bruceae Fructus, was believed to be one of the major active principles responsible for the anticancer effect of Bruceae Fructus. In the present study, pioneering effort was devoted to investigate the *in vitro* and *in vivo* chemosensitizing effect of brusatol toward GEM and 5-FU, and to unravel the potential underlying molecular mechanisms.

We have demonstrated in the present study that brusatol significantly inhibited the proliferation of human PanCa cells in a dose- and time- dependent manner, and brusatol at low concentration was able to significantly inhibit the proliferation of PanCa cells without affecting the viability of normal gastric epithelial cell. Besides, the anti-PanCa effect of brusatol was more potent than that of GEM or 5-FU alone, which also displayed great cytotoxic effect to normal gastric epithelial cell. It was also observed that brusatol showed a synergistic anti-proliferative effect toward both PANC-1 and Capan-2 cell lines when combined with GEM or 5-FU, with the CI values being in the range of 0.2-0.8, indicative of a strong synergistic action. Furthermore, it was found that brusatol monotherapy and combination treatment caused cell cycle arrest at G2/M phase, induced apoptosis, and inhibited several transcription factors and various biomarkers linked to survival, proliferation, and metastasis such as NF-κB p65, Bcl-xL, and PCNA in PanCa cells. These results suggest that brusatol could accentuate the apoptotic effect of GEM or 5-FU by inhibiting anti-apoptotic proteins in PanCa cells. The observed down-regulation of anti-apoptotic proteins was believed to contribute to the synergistic inhibition of PanCa cell growth exerted by the combined treatment of brusatol and GEM or 5-FU.

Based on these promising *in vitro* results, we therefore sought to evaluate the *in vivo* efficacy of brusatol in PanCa growth, using orthotopic models of PanCa derived from the highly malignant PANC-1 and Capan-2 cell lines. Our results suggested that daily brusatol administration for 28 days significantly decreased pancreatic tumor growth in both PANC-1 and Capan-2 cell orthotopic mouse models, without causing significant weight loss, mortality or other noticeable abnormalities on the heart, liver and kidney of the experimental animals. Furthermore, it was also shown that brusatol significantly synergized with GEM/5-FU and inhibited the tumor growth (tumor size, tumor weight and Luc-signal intensity) of mice more significantly than treatment with either single agent, while caused no observable side effects, further supporting the *in vitro* anti-PanCa activity. These findings strongly indicated that the enhanced anticancer efficacy and reduced cytotoxicity could be achieved through optimized combination treatment.

One of the major culprits involved in the development of drug resistance and prognosis of the disease is EMT, which refers to the absence of epithelial phenotype and presence of mesenchymal characteristics. The activation of EMT process is reported to facilitate embryonic development, tissue formation as well as tumor invasion and metastasis [19]. The hallmark of EMT is the loss of the epithelial homotypic adhesion molecule E-cadherin and the gain of mesenchymal markers such as vimentin [20]. In various human cancers, E-cadherin loss is related to poor prognosis, tumor progression and metastasis. Twist is a basic helix-loop-helix (basic Helix-loop-Helix bHLH) transcription factor located in autosomes. Several reports have indicated that Twist is the key regulator of the EMT process associated with tumor cell proliferation, differentiation, metastasis, invasion and anti-apoptotic process, with clinical implication of poor prognosis and metastasis in many kinds of cancers, such as breast, esophageal, gastric and prostate cancer. Twist is also an inhibitor of apoptosis [21-24]. Over expression of Twist often promotes cell colony formation, prevents apoptosis, and induces drug resistance and metastasis of tumor, with a consequence of an increased tumor invasiveness and poor prognosis [25]. In our experiments, brusatol monotherapy or combination treatment significantly increased the expression of E-cadherin and suppressed the expression of vimentin and Twist in both PANC-1 and Capan-2 cells, and the observation was believed to be associated with the inhibition of PanCa growth *in vitro* and *in vivo.*

This is the first report to show that brusatol was able to suppress PanCa growth and enhance the anti-PanCa effect of GEM and 5-FU in an orthotopic mouse model. Based on our promising *in vitro* and *in vivo* results, brusatol could greatly sensitize PanCa to GEM and 5-FU while it exhibited a more favorable safety profile. The result indicated that brusatol might reduce the dose of chemotherapeutic agents while exerting superior beneficial effect for the treatment of PanCa. The synergistic inhibition by brusatol and chemotherapeutic agents observed both *in vitro* and *in vivo*, together with the absence of overt toxicity, strongly indicated that brusatol is a promising adjuvant to current chemotherapy regimen for this deadly human malignancy. However, to corroborate this potential, further experiments should be performed on this model to monitor the mouse survival benefit treated with the combinations.

Taken together, our present work laid a solid foundation for further in-depth studies to evaluate the overall survival benefit, long-term safety, pharm-acokinetics in animal models including the genetically engineered pancreatic cancer mouse model. The results from this work also provided justification for conducting clinical trials in future to evaluate the safety and effectiveness of this natural product on pancreatic cancer. The development of brusatol into an anti-PanCa adjuvant would add new therapeutic dimensions to the current limited approach in the management of this most deadly malignancy in human.

## MATERIALS AND METHODS

### Cell lines and reagents

Human pancreatic cancer cell lines (PANC-1, Capan-1, Capan-2 and SW1990) and non-tumorigenic human gastric epithelial cells were obtained from the ATCC (Manassas, VA, USA). The cell lines were authenticated by short-tandem repeat analysis. Cell lines were initially expanded and cryopreserved within 1 month of receipt and were typically used for 3 months; and at which time, a fresh vial of cryopreserved cells was used (2013-2015). These cells were maintained in culture as an adherent monolayer in DMEM or IMDM supplemented with 10% FBS.

Brusatol (CAS: 14907-98-3) was isolated from Bruceae Fructus in our laboratory, and its structural identity was confirmed by comparing its NMR and HRMS data with those published previously [12]. Its purity was determined to exceed 98% by HPLC analysis. All cell culture reagents were purchased from Invitrogen (Grand Island, NY, USA). Gemcitabine (GEM) (Gemzar, Eli Lilly, USA) and 5-Fluorouracil injection (5-FU) (China Food and Drug Administration, China; approval number H12020959) were procured through the Pharmacy of the Clinical Center, Shenzhen People’s Hospital, Guangdong Province, China.

### Cytotoxicity assay, synergistic effects, cell death detection, apoptosis detection, cell-cycle analysis and plasma-specific enzyme level measurement

The above assays were performed using kits from various manufacturers. Details are provided in the Supplementary Methods.

### Western immunoblotting and real-time PCR

Western immunoblotting technique was used to analyze the expression of Twist, NF-κB, PCNA, Bcl-xl, vimentin and E-Cadherin. Real-time PCR technique was used to analyze the expression of Twist and E-Cadherin. Details are provided in the Supplementary Methods.

### Construction of PanCa cell lines stably expressing CMV-EGFP-linker-Luc

To monitor the *in vivo* PanCa growth, PANC-1 and Capan-2 cells were stably transfected with the luciferase gene. CMV-EGFP-linker-Luc-PGK-Puro-L.V. (constructed by Obio Technology, Shanghai, China) was a lenti-virus and could express green fluorescent protein and luciferase [26]. Lentiviral was transferred into PANC-1 and Capan-2 cells (5 × 10^4^ cell/well in 24-well plates) for a 24 h infection in the presence of polybrene (final concentration 5 μg/mL; Sigma-Aldrich). After 24 h, the culture medium was then replaced by fresh medium. After culture for a further 72 h, cells were incubated with puromycin (final concentration 2 μg/mL) for a period of 14 days for selection. Expression of EGFP was observed by fluorescent microscopy and luciferase expression was confirmed using the Dual Luciferase Assay (Promega), and emitted light was directly proportional to the cell number.

### *In vivo* studies

Male BALB/c nude mice (6 weeks of age) were supplied by the Laboratory Animal Services Centre, CUHK. Animals were bred and maintained in a pathogen-free condition with sterile food and water *ad libitum* in specifically designed air-controlled rooms with a 12-h light/dark cycle. The care and use of the animals were in compliance with the institutional guidelines, and the experimental procedures were approved by the Animal Experimentation Ethics Committee of CUHK (Ref. 14/086/MIS).

Human PanCa cell orthotopic xenograft nude mouse model was established using *in situ* injection method as described previously [27]. Capan-2 and PANC-1 cells, which were stably transfected with EGFP and luciferase as described above, were resuspended in PBS and kept on ice until injection. Male nude mice were anesthetized with ketamine/xylazine (100/10 mg/kg), a small left abdominal flank incision was made, and the pancreas was carefully exposed. Capan-2 (2 × 10^6^ cells) and PANC-1 cells (5 × 10^6^ cells) were injected into the tail of the pancreas with a Hamilton syringe. The pancreas was then returned to the peritoneal cavity, and the abdominal wall and the skin were closed with 6-0 Dexon sutures. One week after tumor transplantation, mice were anesthetized and images of the tumors captured using a Carestream In Vivo Imaging System (MS FX PRO, USA). It was found that all tumors were of similar size. Mice were then randomized into 7 groups of 6 mice each: the control group, GEM group, 5-FU group, two brusatol treatment groups of different dosages (1 or 2 mg/kg), GEM and brusatol (2 mg/kg) combination group, and 5-FU and brusatol (2 mg/kg) combination group.

The tumor-bearing mice were periodically injected *i.p.* with D-luciferin (150 mg/kg, Life Technologies, USA) and anesthetized once a week. After D-luciferin injection (10 min), luminescence was measured using a Carestream In Vivo Imaging System (MS FX PRO, USA), which could be used to obtain X-ray and concurrent bioluminescence images. By combining bioluminescence imaging (BLI) with digital x-ray, the system’s highly improved sensitivity allowed us to precisely locate, identify and monitor tumor morphological changes [27,28]. Quantitative analysis of the optical signal capture was carried out with the Carestream MI software v5.0.5.29 (Carestream Health, Inc., Woodbridge, CT, USA). Fluorescence intensity from subcutaneous and intra-peritoneal tumors was measured by creating an automatic ROI threshold to 30% of each tumor’s maximum intensity and the mean intensity of the area was examined. Fluorescence intensities were normalized to the peak angle of detection, and real-time determination of tumor burden was done by quantifying fluorescent surface area [29]. Mice were imaged on 7, 14, 21, 28 and 35 days after tumor implantation. Treatment was continued for 5 weeks and animals were sacrificed 1 week later. Whole blood was obtained by cardiac puncture. Primary tumors in the pancreas were excised and the final tumor volume was measured as V = (*a×b×c*)/2, where *a* indicates the length, *b* the width, and *c* the depth of tumor [30]. Half of the tumor tissue was formalin-fixed and paraffin-embedded for immuno-histochemistry and routine H&E staining. The other half was snap frozen in liquid nitrogen and stored at −80°C.

### Histology and tissue analysis

Formalin-fixed tissues were embedded in paraffin and cut into 6-μm sections. Sections were evaluated by H&E staining and immunohistochemical analysis using antibodies specific for E-cadherin and Twist. The stained TMA slides were assessed independently by two experienced pathologists in a blinded manner. Each slide was scored semi-quantitatively on the basis of percentage and intensity of the stained normal or neoplastic epithelial cells. The percentages of stained cells were scored using previously described methods [31,32]. Details of histological analysis are provided in the Supplementary Methods.

### Statistical analysis

Results were expressed as mean ± SEM. Data were analyzed by the t test or ANOVA and results were considered significant at *P* < 0.05. A significant interaction was interpreted by a subsequent median effect principle of the Chou-Talalay method.

## SUPPLEMENTARY MATERIALS TABLE


