# Supplementary material for: Exploring brusatol as a new anti-pancreatic cancer adjuvant: biological evaluation and mechanistic studies
Source: Oncotarget. 2017 May 10;8(49):84974–85. doi: 10.18632/oncotarget.17761 (PMC5689587; doi:10.18632/oncotarget.17761)
Supplement: Supplementary file 1 [file oncotarget-08-84974-s001.pdf]

Exploring brusatol as a new anti-pancreatic cancer adjuvant: biological evaluation and mechanistic studies

SUPPLEMENTARY MATERIALS

Cytotoxicity assay

The first-line chemotherapeutic agents, GEM and 5-FU were selected as positive controls. Brusatol was reconstituted in DMSO to produce a stock solution and then diluted with culture medium to various concentrations for cell culture experiments. To minimize the potential solvent effect on cell growth, the final concentrations of DMSO in all culture wells were kept below 0.05%. PANC-1, SW1990, Capan-2, Capan-1 and GES-1 cells were all seeded in 96-well plates at 1×10^4 cells/well. Drug treatment was initiated 24 h after seeding. Cells were incubated for 48 or 72 h with brusatol, GEM or 5-FU, which were diluted with culture medium to various concentrations and added to the cells. Cells treated with medium only served as negative control. After the incubation period, treatment-induced cytotoxicity was assessed by MTT assay using the method described previously [1]. Cell viability was expressed relative to untreated vehicle control. All experiments were done in six parallel wells and repeated three times. The IC_{50} values were calculated using the GraphPad Prism 5.0 software (GraphPad Software Inc., San Diego, CA).

Synergistic effects

Cell growth inhibitions exerted by brusatol, GEM, 5-FU, and their combinations were measured by MTT. Drug interactions between brusatol and GEM, brusatol and 5-FU were assessed at a constant concentration ratio of 1:10, using the combination index (CI), where CI < 1, CI = 1, and CI > 1 indicate synergistic, additive, and antagonistic effect, respectively [2,3]. A CI less than 0.7 denotes that the interactions are highly synergistic [4]. On the basis of the isobologram analysis for mutually exclusive effects, the CI value was calculated based on the absorbance of each group as follows: CI = AB/(A*B). AB is the ratio of the combination groups to the control, whereas A or B is the ratio of the single agent groups to the control group. CI and isobologram for combination treatment were calculated and plotted using the CalcuSyn software (Biosoft, Ferguson, MO, USA).

Cell death detection by ELISA

The amount of cleaved DNA/histone complexes (nucleosomes) in cells was quantified using a Cell Death Detection ELISA^{PLUS} kit per the manufacturer's instructions (Roche Applied Science, Basel, Switzerland). PANC-1 and Capan-2 cells were seeded at 3×10^4 cells/well in 24-well plates and incubated with brusatol, GEM or 5-FU. After 24 h treatment, the cells were trypsinized and pelleted by centrifugation of the plate at 200 × g for 10 min and the supernatant was then aspirated. The cells were resuspended and incubated in 200 µL lysis buffer for 30 min at room temperature. After lysis, the intact nuclei were pelleted by centrifugation at 200 × g for 10 min. Then, 20 µL aliquots of the supernatant (lysate) were transferred to streptavidin-coated wells in a microtiter plate and incubated for 2 h with an immunoreagent containing monoclonal antibodies against histone (biotin labeled) and DNA (peroxidase-conjugated). Each well of the plates was washed three times with 250 µL incubation buffer to remove cell components that were not immunoreactive. ABTS solution (100 µL) was added to each well and the plates were incubated at room temperature on a plate shaker for 15 min. ABTS stop solution (100 µL) was then added immediately to each well. Finally, the amount of colored product, i.e. the immobilized antibody-histone complexes (DNA fragments), in the plate was measured at 405 nm on a microplate spectrophotometer (Fluostar Optima, BMG Labtech, Durham, NC, USA) using ABTS solution as a blank control.

Apoptosis detection by flow cytometry

The ApopNexin™ FITC Apoptosis Detection Kit (Chemicon, Temecula, CA, USA) was used to detect apoptosis by flow cytometry. The estimation procedure was performed according to the manufacturer's instructions. PANC-1 cells were seeded at 3×10^5 cells/well in 6-well culture plates. Brusatol was added and incubated for 24 and 48 h at 37 °C, respectively. Adherent and floating cells were collected after treatment, washed in cold PBS, and centrifuged. Five µL Annexin V-FITC was added to each cell pellet in the dark for 10 min at room temperature. Finally, 100 µL of 1 × Annexin V-buffer was added to each sample and the samples were analyzed by flow cytometry (BD, Accuri C6, Becton Dickinson, USA).

Cell cycle analysis by flow cytometry

PANC-1 cells were cultured in 6-well plates at 3×10^5 cells/well, and collected after exposure for 48 h to brusatol (2 µg/mL), GEM (10 µg/mL), 5-FU (10 µg/mL) and combination treatment. After treatment, cells were washed with ice-cold PBS, and permeabilized with ice-cold (at 4 °C) 75% ethanol for overnight. The

permeabilized cells (1 × 10^6 cells) were suspended in 0.5 mL PI/RNase solution (BD Pharmingen, San Diego, CA, USA) and incubated at room temperature for 30 min in the dark to apply PI stain to cellular DNA. The stained cells were analyzed by a flow cytometer (BD). Data were collected and calculated using the FlowJo software, and the percentage of cells in each cell cycle phase was determined.

Plasma-specific enzyme level measurement

Plasma was collected by centrifugation (3000 × g, 10 min, 4 °C) of the blood sample and stored at -20 °C. Concentrations of alanine aminotransferase (ALT), aspartate aminotransferase (AST), and lactic dehydrogenase (LDH) for assessment of liver damage, creatine kinase (CK) for assessment of heart damage, and creatinine (Cr) for assessment of kidney damage were analyzed as per the manufacturer's instructions (Stanbio Laboratory, USA).

Quantitative real-time PCR analysis for E-cadherin and Twist

The expression of E-cadherin and Twist in the brusatol-treated PANC-1 and Capan-2 cells was examined using quantitative real-time PCR. Briefly, total RNA (0.5 µg) extracted from cells using Trizol reagent (Invitrogen, Carlsbad, CA, USA) was reverse transcribed using the TakaRa PrimeScript™ RT reagent Kit. Real-time PCR was done on the SYBR premix EX Taq™ according to the manufacturer's instructions using an ABI ViiA7 Detection Real-Time PCR system. The sequences of the primer pairs used were listed as follows: E-cadherin forward primer, 5′-TGCCGCCATCGCTTACACCATC-3′; reverse primer, 5′-GGTCAGCAGCTTGAACCACC -AG--3′ (amplicon size, 158 bp); Twist forward primer, 5′-CACCATCCTCACACCTCTGCA-TT-3′; reverse primer, 5′-GCTGATTGGCACGACCTCTTG -AG-3′ (amplicon size, 136 bp); 18S forward primer, 5′-CCTGGATACCGCAGCTAGGA-3′ and reverse primer, 5′-GCGGCGCAAT ACGAATGCCCC-3′ (amplicon size, 122 bp). Each reaction contained 0.5 µL target cDNA template, primers (10 µM) used for the amplification of target gene sequences have been described in detail, and SYBR premix EX Taq™ (TakaRa) mix in a 10 µL final volume. The thermal cycling parameters were set as the following conditions: 95 °C for 1 min; 95 °C for 10 s, 55 °C for 15 s, 72 °C for 20 s (40 cycles), and 72 °C for 1 min. Melting curve analysis was performed to confirm amplification specificity of the PCR products. Quantitative RT-PCR was done at least three times. The results were normalised to the house-keeping gene 18S and expressed as relative mRNA expression level according to the ΔΔ CT method.

Western blotting analysis

PANC-1 and Capan-2 cells were seeded in culture dishes (100 mm^2, 1×10^6 cells/dish). After 24 h incubation, cells were treated with brusatol, GEM and 5-FU singularly or in combination at various concentrations. Treated and control cells were lysed after treatment for 48 h with radioimmunoprecipitation assay buffer [1% Triton X-100, 0.25% sodium deoxycholate, 0.1% SDS, 150 mM NaCl, 5 mM EDTA, and 50 mM Tris–HCl (pH 7.5)] containing protease inhibitor cocktail (Roche Molecular Biochemicals, Switzerland). Then protein concentration was determined using a BCA assay (Bicinchoninic acid kit, Sigma-Aldrich, USA). Equal amounts of cell lysate proteins (25 µg) were electrophoresed by SDS-PAGE for 1.5 h at 110 V. The separated proteins were transferred electrophoretically to PVD membrane for 1 h at 300 mA. The membranes were blocked with 5% (w/v) nonfat dry milk in PBS-T (0.1% v/v Tween-20 in PBS) for 2 h and subsequently incubated overnight at 4 °C with primary antibodies at the following concentrations: mouse anti-Bcl-xL [1:200], mouse anti-PCNA [1:200], rabbit anti-NF-κB p65 [1:200], rabbit anti-E-cadherin [1:1000], rabbit anti-Vimentin [1:2000], rabbit anti-Twist [1:200], then washed with TBST (0.1% v/v Tween20 in TBS) and incubated for 2 h with an appropriate secondary antibody [1:2000]. To confirm equal protein loading, the membranes were subsequently stripped and incubated with monoclonal antibody against β-actin [1:2000], then with a horseradish peroxidase conjugated sheep anti-mouse IgG. Protein bands were visualized by reaction with enhanced chemiluminescence assay kit (GE Healthcare, USA).

Immunohistochemistry and scoring of E-cadherin and Twist

The IHC staining was performed using TMA slides which were deparaffinized in xylene, rehydrated through a graded alcohol series, washed with Tris-buffered saline, and processed using a streptavidin-biotin-peroxidase complex method. For antigen retrieval, TMA slides were boiled by a pressure cooker in 10 mM sodium citrate buffer (pH 6) for 10 min. After quenching of endogenous peroxidase activity, E-cadherin and Twist antibodies were used at a special dilution, i.e. 1:3000 and 1:200 respectively, and the specimens were then incubated with primary antibody overnight at 4°C. The corresponding secondary biotinylated rabbit antibody was used at a special dilution for 30 min at room temperature. Then the slides were washed 3 times in Tris-buffered saline and incubated in streptavidin-horseradish peroxidase (1:100, Dako, Denmark). Chromogenic immunolocalization was performed using 0.05% 3,3-diaminobenzidine tetrahydrochloride. Slides were counter-stained with diluted hematoxylin before dehydration and mounting.

Other tissue cores containing PanCa tissues were used as positive controls. The negative control consisted of normal serum substitution for primary antibody.

The stained TMA slides were assessed independently by two experienced pathologists in a blinded manner. Each slide was scored semi-quantitatively on the basis of percentage and intensity of the stained normal or neoplastic epithelial cells. The percentages of stained cells were scored using previously described methods [5,6]: 0 for no staining; 1 point for < 20%; 2 points for 20-75%; and 3 points for > 75% of cells stained. The intensity of staining was graded on the following scale: 0, negative; 1, low; 2, moderate; and 3, strong intensity. The total score was the product of the scores for the intensity and positive rate of the staining. In this study, a final total histological score of 0-2.5 and >2.5 in E-cadherin expression was considered to be low and high expression, respectively, while a total score > 3 in Twist expression denoted a high level of expression.

REFERENCES

1. Yue GG, Chan BC, Hon PM, Lee MY, Fung KP, Leung PC, Lau CB. Evaluation of in vitro anti-proliferative and immunomodulatory activities of compounds isolated from Curcuma longa. Food Chem Toxicol. 2010; 48:2011-2020.
2. Shimamura T, Royal RE, Kioi M, Nakajima A, Husain SR, Puri RK. Interleukin-4 cytotoxin therapy synergizes with gemcitabine in a mouse model of pancreatic ductal adenocarcinoma. Cancer Res. 2007; 67:9903-9912.
3. Byron SA, Loch DC, Wellens CL, Wortmann, A, Wu J, Wang J, Nomoto K, Pollock PM. Sensitivity to the MEK inhibitor E6201 in melanoma cells is associated with mutant BRAF and wildtype PTEN status. Mol Cancer. 2012; 11:75-90.
4. Thomas M, Finnegan CE, Rogers KM, Purcell JW, Trimble A, Johnston PG, Boland MP. STAT1: a modulator of chemotherapy-induced apoptosis. Cancer Res. 2004; 64:8357-8364.
5. He C, Jiang H, Geng S, Sheng H, Shen X, Zhang X, Zhu S, Chen X, Yang C, Gao H. Expression of c-Myc and Fas correlates with perineural invasion of pancreatic cancer. Int J Clin Exp Pathol. 2012; 5:339-346.
6. Ohuchida K, Mizumoto K, Ishikawa N, Fujii K, Konomi H, Nagai E, Yamaguchi K, Tsuneyoshi M, Tanaka M. The role of S100A6 in pancreatic cancer development and its clinical implication as a diagnostic marker and therapeutic target. Clin Cancer Res. 2005; 11:7785-7793.

Supplementary Table 1: Combination index values for combined treatments of brusatol and chemotherapeutic agents

Cell lines	Chemicals	Combination index values		
		ED50	ED75	ED90
PANC-1	BR + GEM (1:10)	0.71960	0.24070	0.15196
	BR + 5-FU (1:10)	0.23358	0.21820	0.47450
Capan-2	BR + GEM (1:10)	0.26840	0.34680	0.46077
	BR + 5-FU (1:10)	0.57288	0.37080	0.24005
